# Mutational analysis of the *RB1* gene in patients with unilateral retinoblastoma

**DOI:** 10.3389/fmed.2024.1406215

**Published:** 2024-08-21

**Authors:** Yacoub A. Yousef, Mona Mohammad, Laith Baqain, Maysa Al-Hussaini, Mayada Abu Shanap, Hadeel Halalsheh, Jakub Khzouz, Imad Jaradat, Mustafa Mehyar, Iyad Sultan, Ibrahim AlNawaiseh, Munir Shawagfeh

**Affiliations:** ^1^Department of Surgery/Ophthalmology, King Hussein Cancer Centre (KHCC), Amman, Jordan; ^2^Department of Cell Therapy and Applied Genomics (CTAG), King Hussein Cancer Centre (KHCC), Amman, Jordan; ^3^Department of Pediatrics Oncology, King Hussein Cancer Centre (KHCC), Amman, Jordan; ^4^Department of Radiation Oncology, King Hussein Cancer Centre (KHCC), Amman, Jordan; ^5^Department of Anesthesia, King Hussein Cancer Centre (KHCC), Amman, Jordan

**Keywords:** germline mutation, inherited disease, *RB1* gene, retinoblastoma, unilateral

## Abstract

**Purpose:**

Retinoblastoma, a childhood cancer originating in the retina, is primarily attributed to pathogenic RB1 mutations The aim of this study is to conduct a mutational analysis of the RB1 gene in cases of unilateral Retinoblastoma among individuals within the Jordanian population.

**Methods:**

In this study, the peripheral blood of 50 unilateral Rb patients was collected, genomic DNA was extracted, and mutations were identified using Next Generation Sequencing (NGS) analysis.

**Results:**

In this cohort of 50 unrelated patients with unilateral Rb, the median age at diagnosis was eight months (mean, 12 months; range; 2 weeks to 54 months). Twenty-eight (56%) were males, 29 (58%) had the disease in the right eye, 3 (6%) had a positive family history of Rb, and 20 (40%) were diagnosed within the first year of life. *RB1* gene pathogenic mutations were detected in 14 out of 50 (28%) patients, indicating germline disease. Among unilateral non-familial cases, 11 out of 47 (23%) were found to have germline RB1 mutations. Overall, five (36%) of the germline cases had the same mutation detected in one of the parents consistent with an inherited disease (four (80%) were of paternal origin); 3 (60%) of these had affected carrier parent, two (40%) had an unaffected carrier parent. Nine (64%) patients had the nonsense mutation, and six (43%) had the mosaic mutation. The significant prognostic factors for positive genetic testing were positive family history (*p* = 0.018) and age at diagnosis less than 12 months (*p* = 0.03). At a median of 54 months follow-up, two (4%) patients were dead from distant metastasis. The overall eye salvage rate was 44% (*n* = 22/50) eyes; 100% for groups A, B, and C, 60% for group D, and none for group E eyes. There was no correlation between the presence of germline mutation and outcome in terms of eye salvage, metastasis, and survival.

**Conclusion:**

In this study, 28% of patients with unilateral Rb had germline *RB1* mutations, of which 43% were inherited, and one-third presented beyond their first year of life. Therefore, molecular screening is critical for genetic counseling regarding the risk for inherited Rb in unilateral cases, including those with no family history, regardless of the age at diagnosis. However, germline mutations did not appear to significantly predict patient outcomes regarding eye salvage, metastasis, and survival.

## Introduction

Retinoblastoma (Rb) is the most common intraocular malignancy in young children, with around 90% of cases diagnosed before age 5 (1–4). Globally, it affects approximately 1 in every 15,000–20,000 live births, with no differences based on race or gender (2, 3). It is typically diagnosed around 18 months of age, and in about 25–35% of cases, both eyes are affected (3, 5). Rb is a lethal disease, and early diagnosis is known to improve survival (6–10). A recent study in Jordan showed that Rb is the most common intraocular malignancy across all age groups, and the mean age-adjusted incidence was 8.2 cases per million children per year for children aged five years or less (one per 15,620 newborns per year) (11, 12). Compared to developed countries in which 10% of Rb patients have familial disease, 15% of Jordanian Rb patients were found to have familial disease (3, 5, 11).

Retinoblastoma is caused by mutations in a specific gene called the *RB1 gene*, located on chromosome 13 (13). In hereditary cases, one copy of the *RB1* gene has a mutation in the germline, and the other copy has a somatic mutation (a mutation at the cellular level). In contrast, both copies in non-hereditary cases have somatic mutations. The *RB1* gene displays a wide variety of mutations, with over 900 reported ones. Clinically, the hereditary form of the disease is often bilateral, can be passed down through generations in an autosomal dominant manner with a high penetrance (90% or more), and typically starts within the first year of life (14–18). Various techniques, such as quantitative multiplex PCR and sequencing or denaturing high-performance liquid chromatography (DHPLC) along with quantitative multiplex PCR for short fluorescent fragments (QMPSF), can detect these mutations in more than 80% of cases (19, 20). Furthermore, the following generation sequencing analysis (NGS) was reported to have a very high sensitivity (almost 100%) in detecting germline *RB1* mutations in germline Rb patients (21, 22).

All patients with bilateral Rb and 10–15% with unilateral Rb have germline *RB1* mutations (23–27). Thus, identifying the specific *RB1* gene mutation and its inheritance pattern can improve our management of the risk for relatives (28, 29). Genetic testing is commonly used to screen for and identify carriers of *RB1* gene mutations among family members of affected individuals and during pregnancy. This testing helps to manage the disease, improves the vision of affected children through earlier diagnosis and treatment, and reduces the need for extensive ophthalmic examination of family members (29). This study aims to perform a mutational analysis of the RB1 gene using Next Generation Sequencing to investigate RB1 gene mutations in Jordanian patients with unilateral Retinoblastoma. This study will evaluate the implications of these mutations for genetic counseling and prognosis by determining the correlation between RB1 mutations and clinical outcomes, including eye salvage, metastasis, and survival.

## Materials and methods

This retrospective study received ethical approval from the Institutional Review Board of the King Hussein Cancer Center (KHCC). The study cohort comprised 50 unrelated Jordanian patients diagnosed with unilateral Rb. All patients underwent diagnosis and treatment at a single tertiary cancer center in Jordan, KHCC, from January 2013 to December 2022.

### Data collection

Various variables were collected for analysis. These variables included the patient’s age at diagnosis, gender, family history, laterality of the affected eye, stage of Rb according to the International Classification of Retinoblastoma (IIRC) at diagnosis (30), the eye salvage rates, metastasis, and survival.

### Inclusion and exclusion criteria

The eligibility criteria for inclusion in this study encompassed Jordanian Rb patients with a clinical and/or pathological diagnosis of unilateral Rb between 2013 and 2022. Furthermore, inclusion required that they consented to collect blood samples for genetic testing of *RB1* pathogenic mutations. In cases where a positive blood sample was obtained, parents were subsequently offered genetic testing for blood. Patients who have a previous sibling with Rb and those patients whose parents declined consent for their participation in the study were excluded. All patients with bilateral Rb were excluded from this study.

### DNA extraction and *RB1* gene testing

Whole blood samples, comprising 10 mL (5 mL for infants), were obtained from the patient and their parents if needed. The blood samples were collected in either yellow-top ACD tubes or lavender-topped EDTA tubes, and this collection process was conducted at room temperature. Subsequently, these blood samples were promptly dispatched to the molecular diagnostics laboratory, where DNA extraction was performed within less than five days using Gentra Puregene Blood Core kit B (QIAGEN). The genomic DNA was extracted and sequenced at high coverage on an NGS platform to test for *RB1* gene mutation. Variants were identified, and their allele frequencies (AF) were calculated using bioinformatics tools. Mosaicism, the presence of different genotypes within a single individual due to post-zygotic mutations, was classified when variants with an AF had significantly lower than the expected 50% for heterozygous mutations, specifically in the 5 to 35% range. These mosaic variants were confirmed through repeated sequencing, ensuring consistent detection at similar allele frequencies across independent runs.

If no mutations were detected in the *RB1* gene, no more testing was done on the parent’s blood. If a mutation was detected in the *RB1* gene in the patient’s blood, the blood of both parents was tested for the same RB1 gene mutation.

### Methods of mutation detection

Genomic DNA obtained from the submitted sample is enriched for targeted regions using a hybridization-based protocol, and sequenced using Illumina technology. Unless otherwise indicated, all targeted regions are sequenced with ≥50x depth or are supplemented with additional analysis. Reads are aligned to a reference sequence (GRCh37), and sequence changes are identified and interpreted in the context of a single clinically relevant transcript. Enrichment and analysis focus on the coding sequence of the indicated transcripts, 20 bp of flanking intronic sequence, and other specific genomic regions demonstrated to be causative of disease at the time of assay design. Promoters, untranslated regions, and other non-coding regions are not otherwise interrogated. Exonic deletions and duplications are called using an in-house algorithm that determines the copy number at each target by comparing the read depth for each target in the proband sequence with both mean read-depth and read-depth distribution obtained from a set of clinical samples. Markers across the X and Y chromosomes are analyzed for quality control purposes and may detect deviations from the expected sex chromosome complement. Confirmation of the presence and location of reportable variants is performed as needed based on stringent criteria using one of several validated orthogonal approaches (31). Sequencing, Confirmatory sequencing, Fibroblast cell culturing gDNA extraction from skin punch biopsy, and RNA sequencing were performed by Invitae Corporation (1,400 16th Street, San Francisco, CA 94103, #05D2040778).

The following additional analyses are performed if relevant to the requisition. For PMS2 exons 12–15, the reference genome has been modified to force all sequence reads derived from PMS2 and the PMS2CL pseudogene to align to PMS2, and variant calling algorithms are modified to support an expectation of 4 alleles. Suppose a rare SNP or indel variant is identified by this method. In that case, both PMS2 and the PMS2CL pseudogene are amplified by long-range PCR, and the variant’s location is determined by Pacific Biosciences (PacBio) SMRT sequencing of the relevant exon in both long-range amplicons. If a CNV is identified, MLPA or MLPA-seq is run to confirm the variant. If confirmed, both PMS2 and PMS2CL are amplified by long-range PCR, and the identity of the fixed differences between PMS2 and PMS2CL are sequenced by PacBio from the long-range amplicon to disambiguate the location of the CNV. For C9orf72 repeat expansion testing, hexanucleotide repeat units are detected by repeatprimed PCR (RP-PCR) with fluorescently labeled primers followed by capillary electrophoresis. Interpretation Reference Ranges: Benign (normal range): <25 repeat units, Uncertain: 25–30 repeat units, Pathogenic (Full Mutation): > = 31 repeat units. A second round of RP-PCR utilizing a non-overlapping set of primers is used to confirm the initial call for suspected allele sizes of 22 or more repeats. For RNA analysis of the genes indicated in the Genes Analyzed table, complementary DNA is synthesized by reverse transcription from RNA derived from a blood specimen and enriched for specific gene sequences using capture hybridization. After high-throughput sequencing using Illumina technology, the output reads are aligned to a reference sequence (genome build GRCh37; custom derivative of the RefSeq transcriptome) to identify the locations of exon junctions through the detection of split reads. The relative usage of exon junctions in a test specimen is assessed quantitatively and compared to the usage in control specimens. Abnormal exon junction usage is evaluated as evidence in the Sherloc variant interpretation framework. Suppose an abnormal splicing pattern is predicted based on a DNA variant outside the typical reportable range, as described above. In that case, the presence of the variant is confirmed by targeted DNA sequencing.

### Statistical analysis

The analysis of genetics data within this patient’s cohort involved using non-parametric tests. Descriptive statistics were used to measure the central tendency of a dataset (the mean, median, and range). The exact Fisher test, a statistical test used to determine if there are nonrandom associations between two categorical variables, is commonly used when the sample size is small or when the assumptions of other tests, such as the chi-square test, are not met. Because of the small number of samples in this cohort, the exact Fisher test was used to calculate the *p*-value; values of 0.05 or less were considered significant.

## Results

Between January 2013 and December 2022, 50 unrelated children with unilateral Rb were included in the study. The 28 parents of the 14 children with germline disease were genetically tested.

### Demographics, clinical features, and tumor characteristics of the affected children

There were 28 males (56%) and 22 (44%) females; all (100%) patients had unilateral disease, and the median age at diagnosis was eight months (mean, 12 months; range; 2 weeks to 54 months). The total number of affected eyes was 50: 29 (58%) right eye and 21 (42%) left eye. Only three (6%) patients had a family history of Rb in one of the parents. According to the International Classification of Retinoblastoma (ICRB), 40 (80%) eyes were in group D or E at diagnosis (Table 1). The overall eye salvage rate was 44% (*n* = 22/50) eyes; 100% for groups A, B, and C, 60% for group D, and none for group E eyes. Further analysis of stage based on age at diagnosis showed that for those less than one year old; seven (35%) eyes were A, B, C, 10 (50%) eyes were group D, and 3 (15%) eyes were group E. In comparison, in those older than one year, there were three (10%) eyes in groups A, B, and C, 18 (60%) in group D, and 9 (30%) in group E. The predominance of advanced stage at diagnosis (D or E) in patients diagnosed after one year (90% vs. 65%) was noted.

**Table 1:** Demographics, clinical features, and tumor characteristics of 50 unrelated children with unilateral retinoblastoma.

		Total	Salvage	*p* value^@^	Positive	Negative	*p* value^&^
		50	22 (44%)		14 (28%)	36 (72%)	
Gender	M	28 (56%)	12 (43%)	0.54	6 (21%)	22 (79%)	0.343
	F	22 (44%)	10 (45%)		8 (36%)	14 (64%)	
Side	Right	29 (58%)	14 (48%)	0.335	5 (17%)	24 (83%)	0.108
	Left	21 (42%)	8 (38%)		9 (43%)	12 (57%)	
Family history	Yes	3 (6%)	3 (100%)	0.043	3 (100%)	0 (0%)	0.018
	No	47 (94%)	19 (40%)		11 (23%)	36 (77%)	
Age (months)	Less than 12	20 (40%)	10 (50%)	0.341	9 (45%)	11 (55%)	0.03
	More than 12	30 (60%)	12 (40%)		5 (17%)	25 (83%)	
Stage (IIRC)*	A	2 (4%)	2 (100%)	<0.0001	2 (100%)	0 (0%)	0.475
	B	2 (4%)	2 (100%)		1 (50%)	1 (50%)	
	C	6 (12%)	6 (100%)		1 (17%)	5 (83%)	
	D	28 (56%)	12 (60%)*		7 (25%)	21 (75%)	
	E	12 (24%)	0 (0%)		3 (25%)	9 (75%)	
Outcome	Eye salvaged	22 (44%)	–	–	8 (36%)	14 (64%)	0.343
	Primary Enucleation	20 (40%)	–	–	4 (20%)	16 (80%)	
	secondary Enucleation	8 (16%)	–	–	2 (25%)	6 (75%)	
Metastasis	Yes	2 (4%)	–	–	1 (50%)	1 (50%)	0.477
	No	48 (96%)	–	–	13 (27%)	35 (65%)	

*IIRC: staging based on international intraocular retinoblastoma classification (30). ^@^*p* value was calculated using the exact Fisher test. The *p* value in this column tells the chance of eye salvage based on each clinical feature. ^&^*p* value was calculated using the exact Fisher test. The *p* value in this column tells the chance of having positive vs negative genetic testing based on each clinical feature.

At the last available follow-up (median = 54 months), two (4%) were dead: one with pinealoblastoma and one with CNS metastasis. No one in this cohort developed a second malignancy. The significant good prognostic factors for eye salvage were positive family history (*p* = 0.043) and early clinical stage at diagnosis (*p* > 0.0001).

### Genetic testing

#### Results of genetic testing

*RB1* gene pathogenic mutation was detected in the DNA extracted from blood lymphocytes of 14/50 (28%) patients (germline disease). Overall, five (*n* = 5/14; 36%) of germline cases had the same mutation detected in one of the parents (inherited disease); 3 (60%) of these inherited cases had an affected carrier parent, while two (40%) had an unaffected carrier parent. Interestingly, out of 5 inherited cases, paternal origin of the *RB1* pathogenic variant was seen in 4 (80%) cases, while maternal origin was seen only in 1 (20%) case. Once we excluded the familial cases, 11 out of 47 (23%) with non-familial cases had the *RB1* gene pathogenic mutation detected in the DNA extracted from blood. Table 1 summarizes the distribution of germline vs. sporadic cases based on demographics and clinical features. The significant prognostic factors for positive genetic testing were positive family history (*p* = 0.018) and age at diagnosis less than 12 months (*p* = 0.03). There was no correlation between the presence of germline mutation and outcome in terms of eye salvage, metastasis, and survival (Table 1).

### Types of mutations

The most frequent type of RB1 pathogenic variant in germline cases was nonsense (introducing a premature stop codon and producing a truncated and presumably non-functional protein), which was detected in 9/14 (64%). On the other hand, six (43%) patients of the whole group had a mosaic mutation. All those with the mosaic mutation had a negative test in the parents. Deletion (Entire coding sequence) mutations that resulted in a premature stop codon were detected in 2 (14%) patients. One (7%) patient had a frameshift mutation, one (7%) had missense mutation (different amino acid incorporation), and one (7%) had an intronic heterozygous mutation (Table 2). Rare benign variants of unknown significance (VUS) were seen in 2 non-familial patients. There was no significant correlation between tumor stage at diagnosis, and eye salvage rate in correlation with the type of mutation (Table 2).

**Table 2:** Types of pathologic *RB1* gene mutations detected in 14 out of 50 children with unilateral retinoblastoma, and correlation with tumor stage and eye salvage.

Exon	Variant	Zygosity	Mutation type	Stage	Salvage
Exon 18	c.1723C > T (p.Gln575Ter)	Mosaic	Nonsense	E	No
Exon 17	c.1666C > T (p.Arg556Ter)	Mosaic	Nonsense	B	Yes
Exon 10	c.958C > T (p.Arg320Ter)	Heterozygous	Nonsense	A	Yes
Exon 10	c.958C > T (p.Arg320Ter)	Heterozygous	Nonsense	C	Yes
Exon 4	c.409G > T (p.Glu137Ter)	Heterozygous	Nonsense	D	No
Exon 17	c.1654C > T (p.Arg552Ter)	Mosaic	Nonsense	E	No
Exon 21	c.2117G > A (p.Cys706Tyr)	Heterozygous	Missense	D	Yes
Exon 18	c.1568 T > G (p.Leu523Ter)	Mosaic	Nonsense	A	Yes
Exon 10	c.958C > T (p.Arg320Ter)	Mosaic	Nonsense	D	No
Exon 11	c.1072C > T (p.Arg358Ter)	Heterozygous	Nonsense	D	Yes
Deletion (entire coding sequence)		Heterozygous	Deletion	D	No
Deletion (entire coding sequence)		Heterozygous	Deletion	E	No
Exon 18	c.1716delT (p.Ile673LeufsTer38)	Heterozygous	Frameshift	D	Yes
Intron 12*	c.1215 + 2 T > A (Splice donor)	Heterozygous	(Intronic)	D	Yes

There was no correlation between presence of germline mutation and outcome in terms of eye salvage, metastasis, and survival. * *RB1*, Intron 12, c.1215 + 2 T > A (Splice donor), heterozygous, PATHOGENIC. This sequence change affects a donor splice site in intron 12 of the *RB1* gene. It is expected to disrupt RNA splicing. Variants that disrupt the donor or acceptor splice site typically lead to a loss of protein function (32), and loss-of-function variants in *RB1* are known to be pathogenic (5). Disruption of this splice site has been observed in individual(s) with bilateral retinoblastoma (33, 34). In at least one individual the variant was observed to be de novo. It has also been observed to segregate with disease in related individuals. Algorithms developed to predict the effect of sequence changes on RNA splicing suggest that this variant may disrupt the consensus splice site. For these reasons, this variant has been classified as Pathogenic.

## Discussion

Genetic testing to identify *RB1* pathogenic mutations is critical in forecasting disease risk, reducing potential health complications, and facilitating screening of at-risk family members. Rb is an inherently “genetic” form of ocular cancer, and all patients with bilateral Rb and 10–15% of patients with unilateral Rb have germline *RB1* mutations (23–27, 35). Our cohort of 50 unrelated Jordanian patients exhibits a notably higher detection rate of pathogenic mutations in unilateral Rb cases. Among all patients with unilateral Rb in this cohort, 28% were found to have germline *RB1* mutations. Furthermore, 23% of patients with non-familial unilateral Rb had germline *RB1* mutations.

This higher incidence may initially seem due to selection bias, as some patients had a family history of the disease; however, 23% of unrelated patients with a negative family history of Rb had germline disease. This percentage still exceeds the commonly presumed rate of 10–12%. Notably, it surpasses the 5% rate reported in a French study, which used semi-automated denaturing high-performance liquid chromatography (DHPLC) and quantitative multiplex PCR of short fluorescent fragments (QMPSF) methods before the era of NGS (20). Next-generation sequencing (NGS) analysis of the *RB1* gene can detect low-level mosaic variants with a frequency between 8 and 24% in blood DNA (36). In our study, where we used NGS, 6 out of 14 (43%) patients with germline mutations had mosaic mutations, much higher than the previously reported 5.5% mosaic mutations in germline Rb patients (37). This variance in detection rates across studies likely arises from the differences in the methodologies’ sensitivity. Therefore, the actual incidence of germline disease in sporadic unilateral cases may need to be reconsidered at around 23–28% rather than the previously estimated 5–12% (20, 35–38).

Molecular testing in Rb suffered from limited adoption in the past due to the complex nature of *RB1* pathogenic mutations, which are widely distributed across the *RB1* gene, making comprehensive and cost-effective detection historically challenging (39–41). However, recent advancements in NGS have provided a reliable and susceptible method for detecting germline mutations in the *RB1* gene, especially in germline Rb patients (21, 22), compared to prior studies where the detection rate for *RB1* mutations, including bilateral and familial cases, ranged from 19 to 72% (33, 36, 37, 42–48) (75). On the other hand, Whole Genome Sequencing (WGS) is an alternative to NGS that offers comprehensive coverage of both coding and non-coding regions of the *RB1* gene, potentially providing deeper insights into genetic alterations (34, 49, 50). However, it is essential to recognize that WGS comes with inherent challenges, such as higher costs and computational complexities, which may limit its widespread clinical application in retinoblastoma diagnosis. While NGS is a powerful tool for mutation detection, it is not infallible, and false negatives can occur, particularly in cases where deep intronic variants or regulatory regions impacting gene expression are not adequately covered (34, 49–51). This highlights the importance of employing comprehensive genetic testing approaches encompassing the entire genomic landscape of the RB1 gene. Our results are reliable, and using this susceptible technique may explain the high rate of unilateral germline Rb cases in our series. However, although the frequency of *RB1* pathogenic variant mutations varies, the patterns remain remarkably similar across diverse populations. Predominantly, these mutations include nonsense, missense, and frameshift mutations (comprising 60–90% of cases). Our findings align with the literature, with approximately 78% of mutations categorized as nonsense, missense, or frameshift mutations (33, 42–48).

In cases where the *RB1* pathogenic mutation is unknown, all relatives with a higher-than-normal population risk of carrying the mutant allele (1/15,000) necessitate clinical surveillance for early Rb diagnosis. This includes infants and children up to the age of 7 years who undergo examination under anesthesia (EUA) (52). Furthermore, lifetime risk assessment for secondary non-Rb cancers is essential for individuals of all ages (9, 53). When the *RB1* status of the proband is known, the precise status of each relative can be accurately determined. Given that the majority of *RB1* pathogenic mutations are novel or absent in unilateral cases (72% in our study), most relatives will be identified as non-carriers of the *RB1* pathogenic mutation. Offspring of non-carrier parents will have the same Rb risk as the general population. In contrast, offspring of germline patients and carrier parents (even if they do not have Rb) are at a significantly higher risk of developing Rb. Thus, they require more extensive early screening and follow-up (20, 54–56) (75). In this cohort of Jordanian unilateral Rb patients, 72% were non-germline, and around half of those with germline mutation had new mutations, i.e., not hereditary. As a result, each child with unilateral Rb in Jordan carries a 23–28% risk of carrying germline disease and a 12% risk of inheriting the mutation from one of their parents. This translates to a 14% risk for offspring and a 6% risk for siblings, given the autosomal dominant nature of the disease. This supports the need for genetic counseling and monitoring (54). Of interest, two patients in our cohort had inherited the disease from the unaffected parent (the parent with the pathogenic mutation in the blood but had a normal retina). The RB gene is a tumor suppressor gene, and most retinoblastoma families demonstrate autosomal dominant inheritance with almost complete penetrance and high expressivity. However, some families display a different inheritance pattern characterized by reduced penetrance and expressivity (15, 16, 39). The common theme in low penetrance mutations in RB is a reduction in the quantity or quality of cellular pRB activity. An insufficient amount of normal pRB may result from promoter or splice site sequence mutations. In contrast, pRB may be partially disabled by subtle mutations that globally reduce the stability and binding affinity of the protein or that locally perturb semi-essential functions. The tumor suppressor activity of pRB derives from its ability to arrest the cell cycle and induce differentiation. Some low-penetrance mutations appear to compromise preferentially one or the other of these functions, suggesting that regulation of the cell cycle and differentiation may play cooperative roles in tumor suppression by pRB (15).

Patients with hereditary Rb typically receive their diagnosis at an earlier age than those with non-hereditary Rb (36, 57–59). This difference in the age of diagnosis is particularly noticeable among patients with bilateral Rb when *RB1* mutations are detected (27). However, this trend could be more evident for children with unilateral Rb (60), and some previous studies have indicated that the age at diagnosis does not consistently differ between patients with and without a constitutional *RB1* mutation (57, 60, 61). On the other hand, in our study, patients with unilateral Rb who had pathogenic *RB1* mutations were diagnosed younger than those without the mutation. Around two-thirds of those with germline mutation were diagnosed before the age of one year compared to one-third of those with sporadic non-germline disease, which was statistically significant (*p* = 0.03). However, there was no significant correlation between the eye salvage rate and the age at diagnosis or the patient’s genetic status. Nevertheless, stage at diagnosis was the most important prognostic factor for eye salvage, where 100% of groups A, B, and C were successfully salvaged compared to 60% of group D eyes.

Of interest, we noted that 50% of ocular cases diagnosed before 12 months exhibited successful salvage, whereas only 40% of cases diagnosed after this age threshold achieved salvage, albeit the difference was not statistically significant (*p* = 0.341). However, a discernible trend emerged, suggesting a greater propensity for ocular salvage among younger patients. Upon further scrutiny of tumor staging concerning the age at diagnosis, an exciting pattern appeared. Specifically, 65% of cases diagnosed before one year of age presented with advanced intraocular disease (classified as group D or E), in contrast to 90% of cases diagnosed beyond the first year of life. This discrepancy indicates that a significant portion of intraocular tumors initiated their development within the eye during the inaugural year of life but only received a diagnosis later, thus reflecting delayed diagnosis rather than delayed tumor progression.

Furthermore, this observation underscores the apparent independence of the type of germline mutation from the likelihood of achieving ocular salvage. Instead, it emphasizes that the most pivotal prognostic determinant for successful ocular salvage remains the tumor stage at diagnosis. Furthermore, a positive family history of Rb was a significant predictive factor for eye salvage, and this is explained by the awareness of the family about the possibility of this cancer in the eye in this family, and because of the implemented screening program that we had at our institution even before implementing genetic testing (9, 10). As expected, all patients with a positive family history of Rb had germline mutation detected by genetic testing of the blood. The genetic status did not correlate with metastasis and death in our series. One patient who did not have germline mutation passed away with CNS metastasis, and one with familial germline diseases passed away with pinealoblastoma.

In conclusion, this study found that 28% of patients with unilateral Rb (23% of those with a negative family history of Rb) had germline *RB1* mutations. This research is among the few that have extensively characterized these mutations in unilateral cases using NGS. Although patients with germline mutations are usually diagnosed at a younger age, one-third of germline patients presented after their first year. This emphasizes the importance of not limiting genetic testing to very young patients. The relatively high occurrence of germline mutations in unilateral Rb patients highlights the value of genetic testing and counseling for families dealing with Rb, especially considering the effectiveness of Next-Generation Sequencing in detecting these mutations. While the study provides valuable insights into the mutational analysis of the RB1 gene in unilateral retinoblastoma patients in the Jordanian community, it’s essential to acknowledge its limitations, including small sample size, single-center design, which could introduce selection bias and limit the generalizability of the findings to a broader population, and retrospective nature of the study. Addressing these limitations and conducting further research with a larger, more diverse sample and a prospective design could enhance the validity and applicability of the study’s findings.

## Data availability statement

The data presented in the study are deposited in the zenodo repository, accession number 13305115 (https://zenodo.org/records/13305115).

## Ethics statement

The studies involving humans were approved by King Hussein Cancer Center IRB. The studies were conducted in accordance with the local legislation and institutional requirements. The ethics committee/institutional review board waived the requirement of written informed consent for participation from the participants or the participants’ legal guardians/next of kin because informed consent was waived by the IRB for this retrospective non-interventional study. No identifying data were used in this paper.

## Author contributions

YY: Conceptualization, Data curation, Formal analysis, Investigation, Methodology, Resources, Writing – original draft, Writing – review & editing. MoM: Data curation, Formal analysis, Writing – original draft. LB: Data curation, Writing – original draft. MA-H: Conceptualization, Writing – review & editing. MAS: Data curation, Writing – original draft. HH: Methodology, Writing – original draft. JK: Methodology, Writing – original draft. IJ: Resources, Writing – review & editing. MuM: Investigation, Writing – review & editing. IS: Conceptualization, Writing – review & editing. IA: Supervision, Writing – review & editing. MS: Supervision, Validation, Visualization, Writing – original draft, Writing – review & editing.
